# Organizational characteristics, outcomes and resource use in ICUs: the ORCHESTRA Study

**DOI:** 10.1186/cc14684

**Published:** 2015-09-28

**Authors:** Derek C Angus, Fernando Bozza, Jeremy Kahn, Jorge Salluh, Marcio Soares, ORCHESTRA Study Investigators, Pedro Brasil

**Affiliations:** 1 University of Pittsburgh Medical Center, Pittsburgh, PA, USA; 2 D'Or Institute for Research and Education, Botafogo, Rio de Janeiro, RJ, Brazil

## Introduction

Detailed information on organizational factors in ICUs located in emerging countries is scarce.

## Objective

To investigate the impact of organizational factors on patient outcomes and resource use in a large sample of Brazilian ICUs.

## Methods

Retrospective cohort study of 59,483 patients (medical admissions: 39,734 (67 %)) admitted to 78 ICUs (private hospitals, *n *= 67 (86 %); medical-surgical; *n *= 62 (79 %)) during 2013. We retrieved demographic, clinical and outcome data from an electronic ICU quality registry (Epimed Monitor System). We surveyed ICUs using a standardized questionnaire regarding organizational aspects, staffing patterns and process of care. We used multilevel logistic regression analysis to identify factors associated with hospital mortality. Efficient resource use was assessed by estimating standardized mortality rates (SMR) and standardized resource use (SRU) adjusted for the SAPS 3 score.

## Results

Forty (51 %) ICUs had critical care training programs. The median nurse-bed ratio was 0.20 (IQR, 0.15-0.28) and board-certified intensivists were present 24/7 in 16 (21 %) of the ICUs. Routine clinical rounds occurred in 67 (86 %) and daily checklists were used in 36 (46 %) ICUs. The most frequently implemented protocols focused on sepsis management and VAP and CLABSI prevention. Median number of patients per center was 898 (585-1715). SAPS 3 score was 41 (33-52) points. ICU and hospital mortality rates were 9.6 % and 14.3 %. Adjusting for relevant patients' characteristics (SAPS 3 score, admission diagnosis, chronic health status, comorbidities, MV use), case volume and type of ICU, clinical protocols jointly managed by different care providers (OR = 0.23 (95 % CI, 0.08-0.64), *p *= 0.005) were associated with lower mortality. Estimated SMR and SRU were 0.97 (0.72-1.15) and 1.06 (0.89-1.37). There were 28 (36 %) "most efficient" (ICUs with both SMR and SRU median), 11(14 %) "overachieving" (ICUs with low SMR and high SRU) and 11 (14 %) "underachieving" (ICUs with high SMR and low SRU) ICUs. "Most efficient" ICUs were usually located in private hospitals, with step-down units and training programs in critical care. In univariate analyses comparing "most efficient" and "least efficient" ICUs, a graduated nurse-bed ratio >0.25 (OR = 4.40 (1.04-18.60)) was associated with efficient resource use. Daily checklists also tended to be associated with efficient resource use (OR = 2.89 (0.95-8.72), *p *= 0.057).

## Conclusion

In emerging countries such as Brazil, organizational factors are potential targets to improve patient outcomes and efficient resources in ICUs.

